# High Prevalence of Vitamin D Deficiency in Pregnant Women: A National Cross-Sectional Survey

**DOI:** 10.1371/journal.pone.0043868

**Published:** 2012-08-24

**Authors:** Stefanie Vandevijvere, Sihame Amsalkhir, Herman Van Oyen, Rodrigo Moreno-Reyes

**Affiliations:** 1 Department of Public Health and Surveillance, Scientific Institute of Public Health, Brussels, Belgium; 2 Department of Nuclear Medicine, Hôpital Erasme, Université Libre de Bruxelles, Brussels, Belgium; Pennington Biomedical Research Center/LSU, United States of America

## Abstract

An increasing number of studies suggest that vitamin D deficiency during pregnancy is associated with multiple adverse health outcomes in mothers, neonates and children. There are no representative country data available on vitamin D status of pregnant women in Europe. The aim of this study was to estimate the prevalence of vitamin D deficiency among Belgian pregnant women and to assess the determinants of vitamin D status in the first and third trimester of pregnancy. The women were selected via a multi-stage proportionate-to-size sampling design. Blood samples were collected and a questionnaire was completed face-to-face. 55 obstetric clinics were randomly selected and 1311 pregnant women participated in the study. The median serum 25-hydroxyvitamin D [25-(OH)D] concentration was significantly lower in the first trimester (20.4 ng/ml) than in third trimester (22.7 ng/ml). Of all women, 74.1% (95%CI = 71.8–76.5%) were vitamin D insufficient (25-(OH)D <30 ng/ml), 44.6% (95%CI = 41.9–47.3%) were vitamin D deficient (25-(OH)D <20 ng/ml), while 12.1% (95%CI = 10.3–13.8%) were severely vitamin D deficient (25-(OH)D <10 ng/ml). Of all women included, 62.0% reported taking vitamin D-containing multivitamins, of which only 24.2% started taking those before pregnancy. The risk of vitamin D deficiency (25-(OH)D <20 ng/ml) was significantly higher for less educated women and women who reported not going on holidays to sunny climates. The risk of severe vitamin D deficiency (25-(OH)D <10 ng/ml) decreased for women who reported alcohol consumption during pregnancy, decreased with more frequent use of sunscreen lotion and increased for smokers and women who reported preference for shadow. In conclusion, vitamin D deficiency is highly prevalent among pregnant women in Belgium and this raises concerns about the health consequences for the mother and the offspring. A targeted screening strategy to detect and treat women at high risk of severe vitamin D deficiency is needed in Belgium and in Europe.

## Introduction

Vitamin D status is a well-known determinant of bone health [1], [2]. Vitamin D deficiency increases the risk of osteoporosis [3] and fractures [4], while in its most severe form it causes rickets in children [5] and osteomalacia in adults [6]. The ubiquitous presence of vitamin D receptors in most tissues, including the placenta, suggests that vitamin D may have other roles as well. Adequate vitamin D intake is associated with a lower risk of cancer [7], [8], [9], [10], [11], cardiovascular diseases [12], autoimmune diseases [13], neurological disorders [14] and diabetes [15]. In addition, an increasing number of studies suggest that vitamin D deficiency during pregnancy is associated with multiple adverse health outcomes in mothers (gestational diabetes and pre-eclampsia), in neonates (wheezing) and children (low bone mineral density, type-1 diabetes, eczema) [16]–[22]. However there is so far no conclusive evidence about the causality of these relationships, as no randomised controlled trials of vitamin D supplementation with an appropriate assessment of a variety of health outcomes have been carried out to date [21].

Humans get vitamin D (cholecalciferol) from exposure to sunlight, diet and dietary supplements. As few food items contain or are fortified with vitamin D (such as liver, fatty fish, eggs, milk and dairy products, soy milk, butter, margarines), the skin synthesis of vitamin D induced by ultraviolet B radiation (UVB) is the main determinant of vitamin D status in the population [23]. Vitamin D once synthesized in the skin is metabolized into 25-dihydroxyvitamin D [25-(OH)D] in the liver. Due to its longer half-life, 25-(OH)D is considered the best bio-marker of vitamin D status. 25-(OH)D is then metabolized in the kidney by the 1-α hydroxylase to the active steroid hormone 1,25-dihydroxyvitamin D [1,25-(OH)_2_D]. Several modifications of vitamin D metabolism occur during pregnancy.

The expression of 1-α hydroxylase is increased in the kidney and placenta and the concentration of serum 1,25-(OH)_2_D increases in normal pregnancy from the first to the third trimester. The role of 1,25-(OH)_2_D during pregnancy to increase intestinal calcium absorption is since long acknowledged [24].

The cut-off points used to define vitamin D insufficiency and deficiency are not well established and remain controversial. Nevertheless there is a consensus to consider serum 25-(OH)D below 20 ng/ml as inadequately low [25], and some evidence suggests that values higher than 30 ng/ml may be associated with better health outcomes in the adult population. [26], [27]. The uncertainty concerning the optimal serum 25-(OH)D concentration in pregnant women is even higher. As long as the proposed values are not validated in clinical trials the controversy will remain [28].

There is a growing concern about the health consequences of the high prevalence of vitamin D deficiency worldwide among the general population, including pregnant women. The adequacy of the current vitamin D dietary recommendations to reach an optimal vitamin D status during pregnancy has been questioned [24]. Although previous small surveys suggest that vitamin D deficiency among pregnant women is common in Europe [29], [30], there are no reliable country-wide estimates of vitamin D status of pregnant women in European countries. Therefore the aim of this study was to carry out the first national representative random sample survey on vitamin D status in pregnant women in a European country and to assess the determinants of vitamin D status in the first and third trimester of pregnancy.

## Methods

### Ethics Statement

This study was conducted according to the guidelines laid down in the Declaration of Helsinki and all procedures involving human subjects were approved by the medical ethical committee of the Erasme hospital in Brussels. The subjects provided written consent for participation in the study.

### Sampling

The target population of the survey comprised all pregnant women in Belgium during the first and the third trimester of pregnancy in the period from September 2010 to June 2011. The women were selected according to a multi-stage proportionate-to-size stratified sampling design as recommended for studies assessing iodine deficiency [31]. The country was divided into two regions. In each region the obstetric clinics were ordered by province and size based on the number of deliveries during the past year and 60 clusters of 4 clinics were selected per region using systematic sampling in order to have enough replacement clinics in case some refused to participate. Out of these 60 clusters, 30 clusters were randomly selected and within each cluster the first clinic was invited to participate. In each clinic all gynaecologist-obstetricians were invited to participate in order to level out a possible gynaecologist effect. The aim was to include 22 women in each cluster of which 11 in the first trimester and 11 in the third trimester of pregnancy.

### Data Collection

Blood samples were collected from the antecubital vein and a general questionnaire about socio-demographic and socio-economic characteristics, smoking and alcohol consumption during pregnancy and during the 4 weeks prior to the interview, diseases and medication and use of food supplements was completed in a face-to-face interview conducted by the study nurse. Women from Algeria, Egypt, Libya, Morocco, Sudan, Tunisia, and Western Sahara were considered of North African descent. For all women included in the study, body mass index (BMI) was obtained from weight and height recorded by the gynaecologist during the first prenatal consultation in the beginning of the first trimester of pregnancy. First trimester BMI was used as a proxy for prepregnancy BMI for both first and third trimester pregnant women.

### Analysis of Samples

Approximately 5 ml whole blood was collected by venipuncture in a non-heparinized tube. Serum aliquots were then stored at –80C for further analysis. Serum 25-hydroxyvitamin D (25-(OH)D) concentrations were measured by radioimmunoassay (Diasorin, Stillwater, MN, USA).

### Statistical Analyses

The statistical analyses were carried out using STATA 10.1 (StataCorp, Texas, USA). As serum 25-(OH)D is not normally distributed, non-parametric methods were used. The median was used as the measure of central tendency.

Differences between regions, trimesters and age groups were explored using two-sample Wilcoxon rank-sum test or Kruskal-Wallis equality-of-populations rank test.

The odds of having a serum 25-(OH)D concentration lower than 20 ng/ml (vitamin D deficiency) versus a non deficient vitamin D status were estimated through multiple logistic regressions while entering the following variables as predictors in the model: season, age, trimester of pregnancy, region, BMI, smoking behaviour, alcohol consumption, use of vitamin D-containing food supplements, fish consumption, milk and dairy product consumption, education level, ethnicity, parity, exposure to sunlight during weekdays, exposure to sunlight during weekend days, use of sunscreen lotion, use of solarium, shadow or sun preference and sometimes going on holidays to sunny climates (yes/no question). In addition the odds of having a serum 25-(OH)D concentration lower than 10 ng/ml (severe vitamin D deficiency) versus a non severe deficient vitamin D status were estimated through multiple logistic regressions while entering the same predictors in the model.

## Results

**Figure 1:**
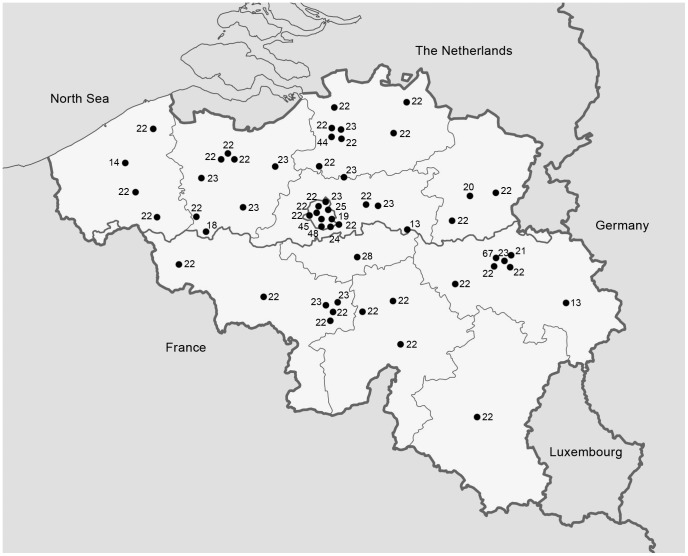
Geographical distribution of the 55 hospitals visited in Belgium and the number of pregnant women (n = 1311) investigated by site (national survey on vitamin D status of pregnant women Belgium 2010–2011).

Among the 1311 pregnant women participating in the survey, there were 271 from Brussels, 597 from Flanders and 437 from Wallonia (Figure 1; Table 1). For 6 women information on the age was missing. The mean age of women was similar in all three regions. For 1307 women a general questionnaire was available. For 1 hospital (n = 23 women) certain questions (mainly nationality, ethnicity, education level) needed to be omitted from the questionnaire upon decision of the ethical committee of this particular hospital.

**Table 1:** Number of pregnant women by region, trimester and age (national survey on vitamin D status among pregnant women Belgium, 2010–2011).

	Regions					
	Brussels**		Flanders**		Wallonia	
Age(years )	1^st^ trimester	3^rd^ trimester	1^st^ trimester	3^rd^ trimester	1^st^ trimester	3^rd^ trimester
15–20	7	2	11	10	21	17
21–25	34	27	77	51	56	73
26–30	41	39	108	149	76	58
31–35	34	53	69	75	49	45
36–40	14	14	23	19	13	24
41–45	2	3	1	3	3	2
Total	132	138	289	307	218	219

6 missing data for age.

** 1 pregnant woman from 2^nd^ trimester.

The characteristics of the pregnant women included in the study are shown in Table 2. More than 50% of the women included had a lower education level, which means only completion of secondary school or even lower education. More than 20% of the women in the sample were from non-Caucasian origin. Of all women included, 15% smoked during pregnancy and 12% reported having drunk alcohol during pregnancy (Table 2).

**Table 2:** Characteristics of the pregnant women included in the study (n = 1311) (Belgian national survey on vitamin D status in pregnant women, 2010–2011).

Characteristic		N
Mean age	28.5±5.1	1305
Mean BMI* (kg/m^2^)	24.4±5.1	1290
% Underweight	5.66	73
% Healthy weight	59.20	763
% Overweight	22.09	285
% Obese	13.10	169
**Ethnicity**		
% White/Caucasian	73.61	965
% Asiatic	2.52	33
% African (Black)	4.88	64
% North African	13.42	176
% Hispanic	0.92	12
% Not known	4.65	61
**Education level**		
% Secondary education or lower	54.54	715
% Higher education	27.23	357
% University or higher	14.87	195
% Other education	0.23	3
% No diploma	0.69	9
% Not known	2.44	32
**Smoking**		
% Yes	16.78	220
% Of which during past 4 wks	15.41	202
% No	82.91	1087
% Not known	0.31	4
**Drinking alcohol**		
% Yes	20.37	267
% Of which during past 4 wks	11.51	151
% No	78.49	1029
% Not known	1.14	15

* First trimester BMI used as a proxy for prepregnancy BMI.

Of all pregnant women included, 640 were in the first, 666 were in the third and 2 were in the second trimester of pregnancy. For 3 women information on the trimester was missing. For 41.7% of the women this was their first pregnancy (unknown for 0.4% of the women), while for 45.6% of the women this would be their first child (status not known for 1.4% of the women). More or less 4% of the women within the sample had had a miscarriage before at least once. For 77.6% of the women their pregnancy was planned (unknown for 3.3% of the women).

The median serum 25-(OH)D concentration in pregnant women was 21.2 ng/mL. The median 25-(OH)D concentration was significantly higher in the first trimester than in the third trimester: 20.4 ng/mL and 22.7 ng/mL respectively. Differences in 25-(OH)D concentration among both regions were not significant. Of all women, 74.1% (95%CI = 71.8–76.5%) were vitamin D insufficient (25-(OH)D concentration <30 ng/ml) and 44.6% (95%CI = 41.9–47.3%) of the pregnant women were vitamin D deficient (25-(OH)D concentration <20 ng/ml), while 12.1% (95%CI = 10.3–13.8%) of the women were severely vitamin D deficient (25-(OH)D concentration <10 ng/ml). The percentage of women with vitamin D insufficiency and deficiency was higher in the first than in the third trimester of pregnancy but the prevalence of severe vitamin D deficiency was higher in the third trimester (Table 3).

**Table 3:** Serum 25-(OH)D concentration in pregnant women (n = 1311) (Belgian national survey on vitamin D status in pregnant women, 2010–2011).

	All women	Wallonia	Flanders	1^st^ trimester	3^rd^ trimester
N	1300	453	633	633	665
Age	28.5±5.1	27.9±5.5	28.8±4.7	28.3±5.1	28.8±5.1
Gestational weeks	22.2±12.5	22.1±12.8	22.4±12.3	9.9±2.8	34.1±3.6
25-(OH)D(ng/ml)					
Median	21.2	20.9	22.3	20.4	22.7*
IQR	13.8–30.0	13.5–29.1	14.6–30.6	13.6–26.7	14.3–34.1
95% CI	20.5–22.2	19.8–22.3	21.2–23.2	19.3–21.1	21.6–24.4
% <10 ng/ml	12.1	12.3	10.9	11.6	12.6
% <20 ng/ml	44.6	45.7	42.3	47.0	42.3
% <30 ng/ml	74.1	76.3	72.5	82.2	66.7

* Different from first trimester pregnant women, p<0.001.

**Figure 2:**
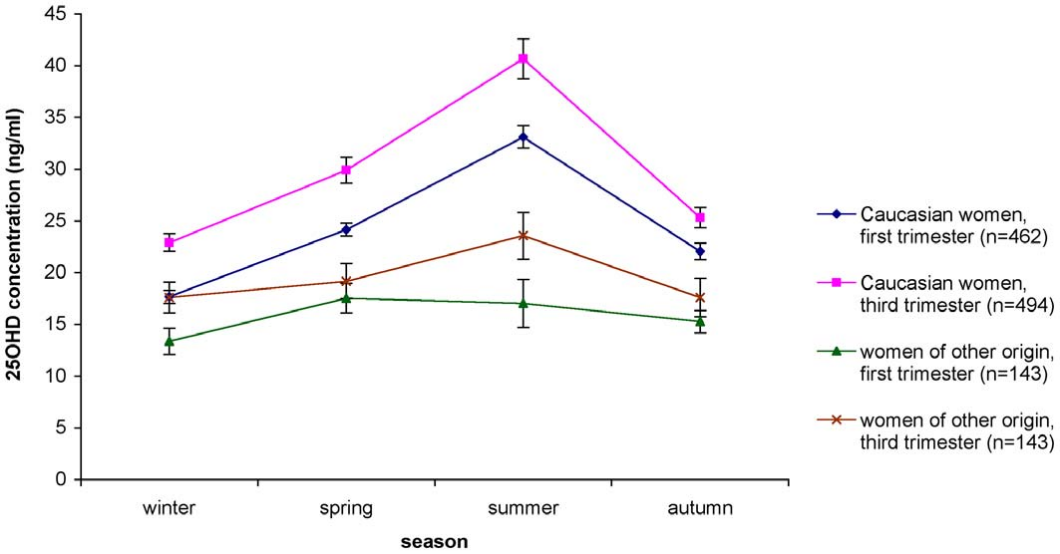
Serum 25-(OH)D concentrations over the different seasons, by trimester of pregnancy and ethnicity (national survey on vitamin D status among pregnant women in Belgium, 2010–2011).

For both first and third trimester women there was a clear seasonal trend in the mean serum 25-(OH)D concentrations with lowest concentrations in winter and highest during spring and summer, while decreasing again in autumn. Women of other ethnic origins were vitamin D deficient (25-(OH)D concentration <20 ng/ml) all year round, except during summer for third trimester women (Figure 2).

Of all women included, 76.2% reported taking at least 1 multivitamin during pregnancy. For 62.0% of them the multivitamin contained vitamin D. In the first and third trimester of pregnancy the percentage of women taking a multivitamin containing vitamin D was 52.6% and 72.3% respectively. Only 24.2% of all pregnant women started taking multivitamins before pregnancy, while 46.6% of these women started taking multivitamins within the first trimester of pregnancy.

The risk of vitamin D deficiency (25-(OH)D <20 ng/ml) increased significantly with BMI and was significantly higher for women who reported not taking vitamin D-containing multivitamins (Table 4). In addition the risk of vitamin D deficiency was threefold higher among women of Asiatic descent, six fold higher for North African women and five fold higher for women of Hispanic descent compared to Caucasians. The risk of vitamin D insufficiency was significantly lower for more educated women and for persons reporting going on holidays to sunny climates. In addition the risk of vitamin D deficiency was significantly lower in summer, spring and autumn compared to winter (Table 4).

**Table 4:** Risk of vitamin D deficiency during pregnancy in Belgium (25-(OH)D <20 ng/ml) (n = 1100), results of multiple logistic regressions.

	N of subjects	N of deficient cases	OR	[95% Conf Interval]		p
**Season**						
Winter	470	278	1.000			
Spring	384	127	0.234	0.165	0.332	<0.001
Summer	93	12	0.100	0.049	0.203	<0.001
Autumn	351	166	0.550	0.391	0.775	0.001
BMI*			1.051	1.022	1.080	<0.001
**Smoking**						
yes	219	118	1.000			
no	1078	465	0.701	0.480	1.025	0.067
**Use of multivitamins containing vitamin D**						
no	489	317	1.000			
yes	809	266	0.224	0.168	0.300	<0.001
**Ethnicity**						
Caucasian, white	955	369	1.000			
Asiatic	33	19	2.823	1.235	6.454	0.014
African (black)	64	34	1.309	0.694	2.468	0.406
North African	174	130	6.048	3.813	9.595	<0.001
Hispanic	12	7	5.305	1.549	18.167	0.008
**Education level**						
Secondary education or less	719	413	1.000			
High school	353	108	0.488	0.349	0.681	<0.001
University	195	52	0.509	0.331	0.783	0.002
**Frequency of fish consumption (non-fatty fish)**						
Never	226	117	1.000			
Less than once a month	225	90	0.564	0.354	0.897	0.016
1–3 days a month	505	216	0.839	0.564	1.248	0.386
1 day per week	270	124	0.794	0.506	1.248	0.318
2–4 days a week or more frequent	69	35	0.562	0.289	1.095	0.090
**Sometimes going on holidays to sunny climates**						
Yes	882	366	1.000			
No	408	215	1.703	1.248	2.324	0.001

* First trimester BMI used as a proxy for prepregnancy BMI.

OR Odds ratio.

BMI Body mass index.

The risk of severe vitamin D deficiency (25-(OH)D <10 ng/ml) was significantly lower among third than first trimester pregnant women (Table 5). In addition risk of severe vitamin D deficiency increased for women who reported not taking vitamin D containing multivitamins, who were of non Caucasian origin and who reported smoking during pregnancy. On the other hand risk of severe vitamin D deficiency decreased for women who reported alcohol consumption during pregnancy. Interestingly, risk of severe vitamin D deficiency decreased with more frequent use of sunscreen lotion and increased for women who reported preference for shadow (Table 5). Exposure to the sun during week and weekend days, and consumption of milk and dairy products were not associated with either severe or normal vitamin D deficiency.

**Table 5:** Risk of severe vitamin D deficiency during pregnancy in Belgium (25-(OH)D <10 ng/ml) (n = 1121), results of multiple logistic regressions.

	N of subjects	N of severe deficient cases	OR	[95% Conf Interval]		p
**Trimester of pregnancy**						
first trimester	633	74	1.000			
third trimester	665	84	1.751	1.128	2.719	0.013
**Season**						
Winter	470	89	1.000			
Spring	384	35	0.283	0.168	0.477	<0.001
Summer	93	0				
Autumn	351	34	0.324	0.191	0.551	<0.001
**Smoking**						
Yes	219	33	1.000			
No	1078	125	0.463	0.268	0.801	0.006
**Alcohol consumption**						
Yes	264	13	1.000			
No	1022	143	2.370	1.162	4.834	0.018
**Use of multivitamins containing vitamin D**						
No	489	115	1.000			
Yes	809	43	0.121	0.075	0.193	<0.001
**Ethnicity**						
Caucasian, white	955	69	1.000			
Asiatic	33	9	6.656	2.413	18.359	<0.001
African, black	64	13	2.605	1.151	5.896	0.022
African, north	174	63	8.174	4.712	14.177	<0.001
**Frequency fatty fish consumption**						
Never	192	18	1.000			
Less than once a month	188	24	2.205	1.010	4.816	0.047
1–3 days a month	498	59	1.481	0.755	2.904	0.254
1 day per week	291	35	1.391	0.671	2.885	0.375
2–4 days a week or more frequent	128	22	1.513	0.659	3.472	0.328
**Use of sunscreen lotion**						
Yes, much of the time	644	36	1.000			
Yes, sometimes	281	34	2.087	1.171	3.722	0.013
No	368	88	3.263	1.935	5.504	<0.001
**Preference sun/shadow**						
Sun	476	53	1.000			
Shadow	497	70	1.983	1.225	3.210	0.005
Does not matter	321	35	1.206	0.691	2.104	0.510

OR Odds ratio.

## Discussion

Despite the fact that more than 60% of the pregnant women reported taking multivitamins containing vitamin D during pregnancy, nearly 45% of the women were vitamin D deficient (25-(OH)D <20 ng/ml). The prevalence of severe vitamin D deficiency was 12% during the first trimester and was slightly higher, 13%, during the third trimester of pregnancy. A previous small study in Brussels suggested that the prevalence of vitamin D deficiency was high among the adult population and that immigrants were at greater risk of vitamin D deficiency [32]. Other small-scale studies in Belgium showed a high prevalence of vitamin D deficiency among Belgian postmenopausal osteoporotic women [33] and elderly [34]. However, the present study is the first national survey on vitamin D status among pregnant women in Belgium. The high prevalence of vitamin D deficiency in Belgium stems from the fact that the contribution of dietary sources to the vitamin D status is negligible as was shown by the Flemish food consumption survey among preschoolers which estimated the mean vitamin D intake at only 2 µg/day [35].

The prevalence of vitamin deficiency (25-(OH)D <20 ng/ml) is high in many European countries [36]–[43] and some studies suggest that pregnant women in Europe are also at high risk of vitamin D deficiency [29], [30]. Similarly as in our pregnant women population, in the adult population the risk of vitamin D deficiency was higher in winter than in summer and increased with BMI [36], [37], [44], [45], [46]. The variations of 25-(OH)D concentration with seasons reflect the changes in UVB exposure, one of the main determinants of vitamin D status in many European countries. The association of vitamin D status with BMI has been attributed to an excessive storage of vitamin D in fat tissue decreasing thereby serum concentrations [47]. Ethnicity was also a major determinant of vitamin D status in the present study, as previously reported in the adult population [32] and in pregnant women [29], [48], [49]. In Belgium, pregnant women of different ethnic origins had substantially lower vitamin D concentrations than Caucasian women and were vitamin D deficient all year long except during summer for third trimester women. In addition education level was associated with vitamin D status in our pregnant women population. Smoking increased the risk of both vitamin D deficiency and severe vitamin D deficiency; the mechanism for this appears to be unclear [50]. Interestingly the risk for severe vitamin D deficiency was lower among women who reported alcohol consumption during pregnancy. The latter has been found also among Korean men [51].

Variables influencing the formation of previtamin D3 in the skin include skin pigmentation and intensity of the solar UV light [52]–[55]. In summer, light-skinned people who spend at least 15 minutes outside during the day with their hands and face exposed will have adequate vitamin D levels. Sunscreen lotions prevent UV radiation from reaching the skin and might therefore reduce the skin’s vitamin D production [56]. However, another study found this effect to be only minor [57].

In Belgium, pregnant women who reported going on holidays to sunny climates had a lower risk of vitamin D deficiency and women who reported using sunscreen lotion had a lower risk of severe vitamin D deficiency. The latter is possibly due to the fact that women using sunscreen lotion are more often exposed to the sun. Women who reported a preference for shadow had a higher risk of severe vitamin D deficiency in our study.

The adequate intake of vitamin D during pregnancy and lactation is unknown, although it appears to be greater than the current dietary recommendations of 400 IU/d or 10 µg/d [58]. Some studies suggest that the dietary requirement during pregnancy and lactation may be as high as 6000 IU/d [59] and recognize that at least 1500–2000 IU/d of vitamin D may be needed in order to maintain a blood level of 25(OH)D above 30 ng/ml [24]. As those authors recognize that the evidence to propose such intakes is scarce and as long as the health benefits of having serum 25(OH)D levels higher than 30 ng/ml are not clearly established particularly in pregnant women, the Belgian Superior Health council still recommends a vitamin D supplement of 20 µg/day or 800 IU during pregnancy [58]. However, the vitamin D content of multivitamins for pregnant women in Belgium is only 400 IU indicating even this recommendation is not followed as pregnant women only take one multivitamin pill a day.

In the absence of survey data from other European countries, we suspect that the prevalence of vitamin D deficiency in Belgium likely reflects the situation in other Western European countries. This assumption is based on the fact that the main risk factors associated with vitamin D deficiency (sun exposure and/or ethnicity) are common to many European countries. In addition to the uncertainty concerning the optimal vitamin D intakes preventing vitamin D deficiency, there exists also a lack of recommendations to treat vitamin D deficient pregnant women.

Even in the last published guidelines the treatment of vitamin D deficient women is not specifically discussed [24]. The uncovering of the magnitude of vitamin D deficiency in pregnant women in Belgium (and Western Europe) should be translated into new research in order to fill the huge knowledge gap concerning the adequate amount of vitamin D to prevent and treat vitamin D deficient pregnant women. In addition, an increasing number of studies suggest that gestational vitamin D deficiency is associated with multiple adverse health outcomes in mothers and children [16]–[22]. Therefore, there is an urgent need of randomised controlled trials of vitamin D supplementation to investigate the maternal and neonatal health benefits of correcting vitamin D deficiency during pregnancy [24].

Given the high prevalence of vitamin D deficiency in pregnant women in Belgium and probably in many European countries, a vitamin D nutrition policy is needed at the country and European level. The current vitamin D recommendations for pregnant women are clearly insufficient to prevent and even more to treat vitamin D deficient pregnant women. Until the adequate treatment of vitamin D deficient pregnant women is established, a safe approach may be to correct vitamin D deficiency by targeting pregnant women at high risk of severe vitamin D deficiency. In addition the current vitamin D content of multivitamins for pregnancy, 400 IU, do not even comply with the current Belgian recommendations of 800 IU per day, therefore a prudent step should at least be to increase the vitamin D content in multivitamins for pregnant women to 800 IU.

In conclusion, despite that more than 60% of the pregnant women reported taking multivitamins containing vitamin D, vitamin D deficiency is highly prevalent among pregnant women in Belgium and up to 12% of pregnant women are severely vitamin D deficient. A targeted screening strategy to detect and treat women at high risk of severe vitamin D deficiency is clearly needed in Belgium and in other European countries. While several observational studies point to correlations between vitamin D insufficiency and maternal and neonatal ill health, experimental evidence from supplementation clinical trials is needed to inform health policy.
